# 非小细胞肺癌侵犯胸膜或胸壁的影像诊断研究进展

**DOI:** 10.3779/j.issn.1009-3419.2025.106.04

**Published:** 2025-03-21

**Authors:** Yue WANG, Zhiyong YUAN

**Affiliations:** 300060 天津，天津医科大学肿瘤医院，国家恶性肿瘤临床医学研究中心，天津市恶性肿瘤临床医学研究中心; Tianjin Medical University Cancer Institute & Hospital, National Clinical Research Center for Cancer,Tianjin’s Clinical Research Center for Cancer, Tianjin 300060, China

**Keywords:** 肺肿瘤, 胸膜, 影像诊断, TNM分期, Lung neoplasms, Pleura, Imaging diagnosis, TNM staging

## Abstract

分期的准确诊断是非小细胞肺癌（non-small cell lung cancer, NSCLC）治疗和预后的基础，其中肿瘤是否累及胸膜或胸壁是评估周围型肺癌分期的重要环节。计算机断层扫描（computed tomography, CT）、磁共振成像（magnetic resonance imaging, MRI）、超声（ultrasound, US）和正电子发射断层扫描（positron emission tomography, PET）等影像技术广泛地应用于判断NSCLC是否有胸膜侵犯，并且已经有越来越多评估NSCLC是否侵犯胸膜及侵犯程度的研究，本文就胸膜受侵的分期和影像诊断标准作一综述，以期为同行对侵犯胸膜或胸壁的精准诊断提供参考。

## Abstract (en)

Accurate staging is the fundamental basis for the treatment and prognosis of non-small cell lung cancer (NSCLC), and whether the tumor involves the pleura or chest wall is a critical aspect in assessing the staging of peripheral lung cancer. Imaging techniques such as computed tomography (CT), magnetic resonance imaging (MRI), ultrasound (US) and positron emission tomography (PET) are widely used to determine pleural invasion in NSCLC. There has been an increasing number of studies evaluating whether NSCLC invades the pleura and the extent of such invasion. This article provides a review of the staging and the imaging diagnostic criteria of pleural invasion, aiming to offer references for peers in the precise diagnosis of pleural or chest wall invasion.

肺癌在全球恶性肿瘤发病率中位列第二，非小细胞肺癌（non-small cell lung cancer, NSCLC）是其中最常见的病理类型^[1]^。在可手术治疗的NSCLC患者中壁层胸膜或胸壁受侵占5%-8%^[2]^。肿瘤原发灶-淋巴结-转移（tumor-node-metastasis, TNM）对于恶性肿瘤患者预后的影响最为显著。根据第八版国际抗癌联盟（Union for International Cancer Control, UICC）和美国癌症联合委员会（American Joint Committee on Cancer, AJCC）的肺癌TNM分期^[3]^，肿瘤直径<3 cm时，若肿瘤侵犯了脏层胸膜，T分期将会从T1升级至T2，TNM分期也会从IA期升至IB期。Eriguchi等^[4]^发现胸膜（包括叶间胸膜）受累是复发的独立风险因素。因此，影像诊断胸膜或胸壁受累的准确性至关重要，其不仅影响着分期诊断，还对患者整体治疗方案的制定、治疗方式和预后的评估起着关键作用。鉴于精确诊断周围型肺癌胸膜受累及其受累程度对于疾病的全程治疗均有获益，本文就NSCLC侵犯胸膜或胸壁的影像学诊断的进展进行综述。

## 1 肿瘤侵犯胸膜及胸壁的概述

### 1.1 胸膜及胸壁受侵的临床及病理分期

根据第八版肺癌TNM分期系统，除非其他标准赋予更高的T分期，肿瘤侵犯脏层胸膜应分为T2，累及壁层胸膜及胸壁则归为T3^[3]^。在病理诊断方面，Dail等^[5]^提出了细化标准，其研究将胸膜受累分为4期，相应的T分期为：PL0对应T1，PL1或PL2对应T2，PL3对应T3。

### 1.2 跨叶型（adjacent lobe invasion, ALI）NSCLC的分期

#### 1.2.1 关于ALI的概述

叶间胸膜作为脏层胸膜的一种，若肿瘤生长导致叶间胸膜受累并侵犯生长至相邻肺叶，就将被定义为ALI，其在可手术的肺癌患者中占比约为17%^[6]^。

#### 1.2.2 关于ALI分期的讨论

早期，大部分学者同意将ALI归为T2期。例如Miura^[7]^和Nonaka^[8]^等的研究均表明叶间胸膜受侵患者的预后明显优于壁层胸膜受侵的患者（T3），且与脏层胸膜受侵的患者（T2）相似。但之后的大多数研究^[6,9⇓-11]^均建议将ALI的分期进行升级。在第八版肺癌分期^[3]^中，根据肿瘤大小对T分期进行了细分，但仍未对ALI进行单独说明。2017年Liu等^[12]^发现ALI组的生存预后与T3组相近[P=0.93，风险比率（hazard ratio, HR）=1.10，95%CI: 0.69-1.37]，明显高于T2a（HR=2.34, 95%CI: 1.50-3.39）和T2b（HR=1.71, 95%CI: 1.19-2.76）的患者。

基于叶间胸膜存在分化不完全的情况，Ohtaki等^[13]^将ALI细分为ALI-A（ALI-across the interlobar fissure, ALI-A）型和ALI-D（ALI-direct beyond the interlobar fissure, ALI-D）型，并且数据表明二者的生存期有差异，5年总生存期（overall survival, OS）为52.0% vs 85.7%（P=0.010）。ALI-A型的5年OS与PL1-2相似，ALI-D型的5年OS近似于PL0，而且ALI-A型比ALI-D型发生淋巴结转移的概率更高。

ALI的分期一直存在争议（表1），目前来看，多数学者支持将ALI特别是ALI-A型归为T3期；而关于ALI-D型的分期归属仍需更多大样本研究加以验证。

**表1:** 跨叶型NSCLC分期的临床研究汇总

Author	Country	Group	Surgery	5-year OS (%)	N0	N1	N2&up	ALI staging
Demir et al. (2007)^[6]^	Turkey	ALI: 60 T2: 152 T3: 139	10LPR, 10biL, 40P 2W, 115L/LPR/biL, 3P 3W, 61L/LPR/biL, 75P	36.0 56.0 40.0	23 64 63	29 67 49	8 21 27	T3
Miura et al. (1998)^[7]^	Japan	ALI: 18 VPI: 30 PPI: 63	10LPR, 4biL, 4P - -	34.2 42.0 13.6	8 17 20	6 4 18	4 9 25	T2
Nonaka et al. (2005)^[8]^	Japan	ALI: 50 T2: 95 T3: 34	13LPR, 12bil, 23P, 2T - -	63.0 57.0 31.0	27	12	11	T2
Dziedzic et al. (2016)^[9]^	Poland	ALI: 164 T2: 7729 T3: 396	48biL, 53L, 54P, 9sub-L 371biL, 6268L, 518P, 572sub-L 14biL, 64L, 22P, 56sub-L	51.0 68.4 62.9	164 7729 396	- - -	- - -	T3
Haam et al. (2012)^[10]^	Korea	ALI: 46 T2: 590 T3: 201	1W, 4L, 3LPR, 11biL, 27P 2W, 1S, 68PLR, 44L, 75biL 2W, 124L, 16biL, 59P	53.0 68.0 49.0	46 590 201	0 0 0	0 0 0	T3
Kanzaki et al. (2012)^[11]^	Japan	ALI: 25 T2: 682 T3: 294	- - -	31.1 52.0 36.3	14 375 139	4 170 66	7 137 89	T3
Liu et al. (2017)^[12]^	China	ALI: 53 T2a: 106 T2b: 106 T3: 106	13P, 19biL, 14L, 7sub-L 18P, 21biL, 469L, 239sub-L 38P, 14biL, 287L, 106sub-L 54P, 31biL, 395L, 116sub-L	38.6 62.4 54.6 35.8	41 633 345 422	12 114 100 174	0 0 0 0	T3
Ohtaki et al. (2013)^[13]^	Japan	ALI-A: 72 ALI-D: 18 PL0: 1387 PL1-2: 501 PL3: 119	14P, 19biL, 36L+W, 3L+S 2P, 3biL, 9L+W, 4L+S - - -	49.8 76.6 76.6 49.8 37.9	26 6 - - -	23 11 - - -	23 1 - - -	ALI-A: T2b ALI-D: Not upstaging

NSCLC: non-small cell lung cancer; ALI: adjacent lobe invasion; VPI: visceral pleural invasion; PPI: parietal pleural invasion; LPR: lobectomy plus partial resection; biL: bilobectomy; L: lobectomy; P: pneumonectomy; S: segmentectomy; T: thoracotomy; W: wedge resection; sub-L: sub-lobe resection; ALI-A: ALI across the interlobar fissure; ALI-D: ALI beyond the incomplete fissure.

## 2 不同影像技术在诊断肿瘤侵犯胸膜中的应用

计算机断层扫描（computed tomography, CT）是目前肺癌分期的首选诊断方法。由于磁共振成像（magnetic resonance imaging, MRI）软组织对比度更好，所以它对评估胸膜壁层侵犯可能更有优势^[14,15]^。超声（ultrasound, US）^[16,17]^和正电子发射断层扫描（positron emission tomography, PET）^[18]^也在诊断胸膜受累中有所应用。由于肺是不断进行呼吸运动的，所以配合呼吸运动的影像检查^[19⇓⇓⇓-23]^可能会提供更高的诊断效能。关于诊断NSCLC是否侵犯胸膜或胸壁的影像学方法的文献汇总见表2^[15,16,19,21⇓⇓⇓⇓⇓⇓⇓⇓⇓⇓⇓⇓⇓-35]^。因为X线平片对胸膜及胸壁结构难以辨别，所以X线平片目前还未应用于胸膜受侵的诊断。近些年来，随着人工智能算法的发展，影像组学作为其中的佼佼者展示出了其在诊断胸膜受累方面的优势。

**表2:** 关于诊断NSCLC是否侵犯胸膜或胸壁的影像学方法的文献

Author	Patients (All/Invasion)	Method	Parameter	Results in (%) sens/spec
Higashino et al. (2005)^[15]^	60/7	Routine CT Thin-section CT Thin-section sagittal MPR Thin-section coronal MPR	Contact, angle, fat plane	Routine CT: 43/91 Thin-section CT: 71/94 Thin-section sagittal MPR: 86/96 Thin-section coronal MPR: 71/94
Sugama et al. (1988)^[16]^	65/39	US	Infiltration	77 accuracy
Murata et al. (1994)^[19]^	9/2	CT ED CT	Tumor movement	CT: 100/50 ED CT: 100/100
Yokoi et al. (1991)^[21]^	27/7	Pneumothorax CT	Air space	100 accuracy
Kajiwara et al. (2010)^[22]^	100/27	Cine MRI	Tumor movement	100/69
Kodalli et al. (1999)^[23]^	23/11	The inspiratio-expiration MRI	Tumor movement	Middle or basal segments: 100/100 Upper or superior segments: 45 accuracy
Glazer et al. (1985)^[24]^	47/15	CT	Angle, thickening, contact, fat plane	87/59
Imai et al. (2013)^[25]^	169/101	CT	A/D>0.9	CT: 93/95
Ebara et al. (2015)^[26]^	201/61	3D CT	Pleural patterns Rarea>13.4	77 accuracy 89/75
Pennes et al. (1985)^[27]^	33/4	CT	Pleural thickening, Abnormality of extra-pleural fat, Asymmetry of thoracic wall soft tissue, Mass extending into chest wall soft tissues, Rib destruction	100/44 38/92 0/96 12/93 12/96
Uhrmeister et al. (1999)^[28]^	41/23	HR CT SD CT	Contact area, angle, fat plane, infiltration	HR CT: 81/79 SD CT: 96/78
Kuriyama et al. (1994)^[29]^	42/17	3D CT	Pleural thickening or puckering	82/76
Chen et al. (2023)^[30]^	284/101	CT Iodine uptake	Contact length >10 mm, spiculation sign	AUC: 0.73
Lennartz et al. (2019)^[31]^	84/44	Dual-energy CT		
Iizuka et al. (2019)^[32]^	138/64	Risk scoring system	Tumor diameter in CT ≥24 mm, Tumor contact length with pleura in CT ≥16 mm, Smoking index ≥400, Clinically lymph node positive, Tumor with cavity in CT, Serum CEA level >4.4 ng/mL	AUC: 0.68
Usuda et al. (2019)^[33]^	43	DWI	ADC	-
Suzuki et al. (1993)^[34]^	120/19	CT/US	CT: angle, contact, thickening US: disruption, extension, fixation	CT: 68/66 US: 100/98 CT+US: 98/67
Yuan et al. (2018)^[35]^	327	Radiomics signature	Percentile 10%, WavEnLL_S_2, S_0_1_SumAverage	90.6%/93.2%

CT: computed tomography; US: ultrasound; Sens: sensitivity; Spec: specificity; ED CT: multisection expiratory dynamic CT; HR CT: high-resolution algorithm CT; SD CT: soft detail algorithm CT; DWI: diffusion-weighted imaging; ADC: apparent diffusion coefficient; AUC: area under the curve; CEA: carcinoembryonic antigen.

### 2.1 CT

#### 2.1.1 肿瘤侵犯胸膜壁层或延伸至胸壁

截至目前为止，PL3是大多数研究关注的焦点。目前关于壁层胸膜或胸壁的诊断标准是基于Glazer等^[24]^的研究所建立的，分别是与肿瘤接触的胸膜增厚、肿瘤与胸膜接触面>3 cm、肿瘤与胸膜接触面夹角为钝角，其诊断的准确性、灵敏度和特异度分别为56%-89%、38%-90%和40%-96%^[24⇓-26]^。除此之外，还包括胸膜接触面脂肪层消失、胸壁软组织受累、骨质破坏，这也在Pennes等^[27]^的研究中得以证明，并且他们发现了CT诊断胸壁软组织受累灵敏度低和特异度高。为了提高软组织识别的灵敏度，Uhrmeister等^[28]^对病变部位行薄层CT（层厚为1 mm）扫描，并进行了边缘增强算法CT（high-resolution algorithm CT, HR CT）和边缘模糊算法CT（soft detail algorithm CT, SD CT）分析，发现SDCT诊断的灵敏度最高，可以分辨出脂肪层的细微变化。

Kuriyama等^[29]^发现胸膜皱褶即胸膜局部增厚并向肿瘤方向牵拉，诊断胸膜受累的准确性为79%。在胸膜皱褶的不同类型中Ebara等^[26]^发现裙状模式的诊断准确度最高（77%），与Imai等^[25]^观点不同的是，他们认为提出Rarea即构成界面的体素的体积和y轴的体素大小的商与肿瘤大小的比值是预测壁层胸膜受累的独立预测因子，而A/D肿瘤与胸膜接触的界面长度跟肿瘤大小的比值与胸膜受累无关。当Rarea>13.4时，其诊断准确性为77%，灵敏度为89%，特异度为75%。Chen等^[30]^提出肿瘤与胸膜接触长度>10 mm（P=0.001）和毛刺征（P=0.033）是独立预测因子。

与在静态图像上观察肿瘤与胸膜的特征不同，在呼吸运动中观察肿瘤与胸膜关系的动态变化能够提高诊断的准确性。例如，气胸CT^[21]^和多段呼气动态CT^[19]^发现动态CT对胸膜受侵的诊断准确性为100%，但其对胸膜粘连的原因不能明确。双能CT已经证明其对胸膜病变的定性和定量判断的优势^[31,36]^，但在临床的应用仍需更多样本量验证。

#### 2.1.2 肿瘤侵犯脏层胸膜

胸膜标签即肿瘤表面的肺小叶间隔增厚延伸至胸膜表面所形成一条或多条的线状束，是诊断脏层胸膜受累的诊断标准。其中II型胸膜标签即在胸膜端有软组织成分在预测脏层胸膜受侵的准确性最高，为71%，其灵敏度为36.4%，特异度为92.8%^[37]^。除此之外，肿瘤距胸膜的最小距离^[38]^和纵隔淋巴结肿大^[39]^与脏层胸膜受累可能相关。对于亚实性肺结节，Ahn等^[40]^发现实性成分>50%是侵犯脏层胸膜的独立危险因素。Iizuka等^[32]^通过结合临床症状和CT特征提高了模型预测的准确性，其构建的模型受试者工作特征曲线下面积（area under the curve, AUC）值为0.68。

#### 2.1.3 侵犯胸膜弹力层

因为难以在影像上对于PL1和PL2进行区分鉴别，目前仍未有PL1的影像诊断标准，仅能通过病理检查进行确诊。Adachi等^[41]^的研究表明PL1和PL2患者的生存期相差无几，但Hung等^[42]^研究报道，脏层胸膜受累（PL2）是NSCLC患者复发和生存期较差的重要预后因素，仍需研究分析PL1和PL2的预后差异。

### 2.2 MRI

MRI可以评估胸壁侵犯的程度^[43]^，胸壁软组织受累，在T1呈现中等信号、T2呈现高信号^[44]^，且在矢状面的薄层图像的准确性更高^[15]^。扩散加权磁共振成像（diffusion-weighted imaging, DWI）可以从形态学和定性上评价胸膜侵犯及肿瘤良恶性^[33]^。

与动态CT相似，有研究通过观察与胸膜相邻的肿瘤有无呼吸相移（移动超过1/3椎体，或超过5 mm）来评价肿瘤与胸膜的关系。例如，Akata等^[45]^研究报道呼吸动力学MRI（respiratory dynamic MRI, RD MRI）检查的灵敏度为100%，特异度为82.8%。但对于上叶和下叶背段的NSCLC患者，Kodalli等^[23]^发现是否存在呼吸相移的诊断准确性仅为45%，这可能是由于位于上叶的肿瘤活动度小，呼吸相移的诊断准确性会降低。

### 2.3 US

US对于肺癌侵犯胸膜的准确性为77%，其中uP-3（肿块回声延伸至胸壁，存在脏层、壁层胸膜切断征，呼吸时肿块无运动）的准确度为100%^[16]^。与CT检查相比，US诊断肿瘤侵及胸壁的灵敏度和特异度都更高^[17,34]^。目前US的新技术，如经胸US和袖珍式US还未在胸膜受侵的诊断中有所应用。

### 2.4 PET

PET可以提供全身肿瘤组织的代谢信息，帮助排除转移和识别定位可能受累的淋巴结^[46]^。PET/CT通过整合CT的解剖学信息和PET的生物学信息，提高了诊断准确性（可达84%）^[18]^。但由于容积效应的影响，PET所提供的肿瘤胸膜连接处最大标准摄取值（maximum standardized uptake value, SUVmax）值并不能准确反映胸膜是否受累^[47]^。

### 2.5 影像组学

目前已有研究根据提取影像组学特征来诊断胸膜受侵，Yuan等^[35]^应用影像组学预测≤3cm肺腺癌侵犯脏层胸膜的准确性为90.5%，并且percentile10%和wavEnLL_S_2, S_0_1_SumAverage是独立危险因素。但此项研究只局限于I期肺腺癌，有待更大样本量的研究加以验证。

## 3 影像诊断的问题与挑战

目前缺乏关于PL1和PL2预后差异的研究，而且二者没有明确的影像学诊断区别，多数是通过弹力蛋白染色来进行病理诊断，仍需继续探索研究。近年来，虽然壁层胸膜或胸壁受侵的影像学诊断标准目前基本已达成共识，但对于具体CT表现的诊断效能都还有待提高。例如，对于肿瘤与胸膜形成夹角的研究目前只局限于区分锐角和钝角^[26]^，关于是否是角度越大侵犯胸膜的可能性越大等问题还尚不清楚；涉及到肿瘤与胸膜接触界面长度时，不同的研究^[25,26]^的长度界值不尽相同，这可能与纳入患者特征有关。Imai等^[25]^研究的大多是T2和T3期的患者，Ebara等^[26]^纳入研究的患者大都是T1和T2期，所以仍需研究来进一步提高各项CT诊断指征的准确性。

辨别相邻结构改变的性质，对粘连部位进行穿刺活检是金标准，但其存在有创操作的隐患，是否可以通过结合PET的SUV来加以判断，目前尚无相关研究，高分辨率、高对比度的图像开发可能会提高读片的准确性。

影像组学联合CT特征的诊断准确性更高，但由于目前研究选择的病例类型等方面较为局限，有待大样本、多中心研究加以验证。MRI功能成像等技术是否能应用于胸膜诊断目前还未有研究证实。

总之，通过CT成像诊断胸膜或胸壁侵犯的标准目前比较明确。但对于ALI的分期、不同成像技术的局限性、各种影像表现的细化和新技术及人工智能的应用等诸多问题仍有待解决。

## References

[1] Rosell R, Karachaliou N. Large-scale screening for somatic mutations in lung cancer. Lancet, 2016, 387(10026): 1354-1356. doi: 10.1016/S0140-6736(15)01125-3 26777918

[2] McCaughan BC, Martini N, Bains MS, et al. Chest wall invasion in carcinoma of the lung. Therapeutic and prognostic implications. J Thorac Cardiovasc Surg, 1985, 89(6): 836-841. 2987619

[3] Detterbeck FC, Boffa DJ, Kim AW, et al. The eighth edition lung cancer stage classification. Chest, 2017, 151(1): 193-203. doi: 10.1016/j.chest.2016.10.010 27780786

[4] Eriguchi T, Takeda A, Tsurugai Y, et al. Pleural contact decreases survival in clinical T1N0M0 lung cancer patients undergoing SBRT. Radiother Oncol, 2019, 134: 191-198. doi: 10.1016/j.radonc.2019.02.005 31005215

[5] Dail DH, Hammar SP, Colby TV. Pulmonary pathology-tumors. Springer: New York, 1995.

[6] Demir A, Gunluoglu MZ, Sansar D, et al. Staging and resection of lung cancer with minimal invasion of the adjacent lobe. Eur J Cardiothorac Surg, 2007, 32(6): 855-858. doi: 10.1016/j.ejcts.2007.09.017 17936002

[7] Miura H, Taira O, Uchida O, et al. Invasion beyond interlobar pleura in non-small cell lung cancer. Chest, 1998, 114(5): 1301-1304. doi: 10.1378/chest.114.5.1301 9824005

[8] Nonaka M, Kataoka D, Yamamoto S, et al. Outcome following surgery for primary lung cancer with interlobar pleural invasion. Surg Today, 2005, 35(1): 22-27. doi: 10.1007/s00595-004-2894-2 15622459

[9] Dziedzic D, Rudzinski P, Langfort R, et al. Results of surgical treatment and impact on T staging of non-small-cell lung cancer adjacent lobe invasion. Eur J Cardiothorac Surg, 2016, 50(3): 423-427. doi: 10.1093/ejcts/ezw110 27032471

[10] Haam SJ, Park IK, Paik HC, et al. T-stage of non-small cell lung cancer directly invading an adjacent lobe. Eur J Cardiothorac Surg, 2012, 42(5): 807-810; discussion 810-811. doi: 10.1093/ejcts/ezs171 22723615

[11] Kanzaki R, Ikeda N, Okura E, et al. Surgical results and staging of non-small cell lung cancer with interlobar pleural invasion. Interact Cardiovasc Thorac Surg, 2012, 14(6): 739-742. doi: 10.1093/icvts/ivs094 22422874 PMC3352743

[12] Liu M, Wigle D, Wampfler JA, et al. T category of non-small cell lung cancer invading the fissure to the adjacent lobe. J Thorac Cardiovasc Surg, 2017, 154(5): 1777-1783.e3. doi: 10.1016/j.jtcvs.2017.07.069 29042049 PMC5688515

[13] Ohtaki Y, Hishida T, Yoshida J, et al. The clinical outcome of non-small cell lung cancer patients with adjacent lobe invasion: the optimal classification according to the status of the interlobar pleura at the invasion point. Eur J Cardiothorac Surg, 2013, 43(2): 302-309. doi: 10.1093/ejcts/ezs268 22593185

[14] Hollings N, Shaw P. Diagnostic imaging of lung cancer. Eur Respir J, 2002, 19(4): 722-742. doi: 10.1183/09031936.02.00280002 11999004

[15] Higashino T, Ohno Y, Takenaka D, et al. Thin-section multiplanar reformats from multidetector-row CT data: utility for assessment of regional tumor extent in non-small cell lung cancer. Eur J Radiol, 2005, 56(1): 48-55. doi: 10.1016/j.ejrad.2005.04.002 16168264

[16] Sugama Y, Tamaki S, Kitamura S, et al. Ultrasonographic evaluation of pleural and chest wall invasion of lung cancer. Chest, 1988, 93(2): 275-279. doi: 10.1378/chest.93.2.275 3276452

[17] Bandi V, Lunn W, Ernst A, et al. Ultrasound vs CT in detecting chest wall invasion by tumor: a prospective study. Chest, 2008, 133(4): 881-886. doi: 10.1378/chest.07-1656 17951616

[18] Lardinois D, Weder W, Hany TF, et al. Staging of non-small-cell lung cancer with integrated positron-emission tomography and computed tomography. N Engl J Med, 2003, 348(25): 2500-2507. doi: 10.1056/NEJMoa022136 12815135

[19] Murata K, Takahashi M, Mori M, et al. Chest wall and mediastinal invasion by lung cancer: evaluation with multisection expiratory dynamic CT. Radiology, 1994, 191(1): 251-255. doi: 10.1148/radiology.191.1.8134583 8134583

[20] Shirakawa T, Fukuda K, Miyamoto Y, et al. Parietal pleural invasion of lung masses: evaluation with CT performed during deep inspiration and expiration. Radiology, 1994, 192(3): 809-811. doi: 10.1148/radiology.192.3.8058952 8058952

[21] Yokoi K, Mori K, Miyazawa N, et al. Tumor invasion of the chest wall and mediastinum in lung cancer: evaluation with pneumothorax CT. Radiology, 1991, 181(1): 147-152. doi: 10.1148/radiology.181.1.1887024 1887024

[22] Kajiwara N, Akata S, Uchida O, et al. Cine MRI enables better therapeutic planning than CT in cases of possible lung cancer chest wall invasion. Lung Cancer, 2010, 69(2): 203-208. doi: 10.1016/j.lungcan.2009.10.016 19945190

[23] Kodalli N, Erzen C, Yuksel M. Evaluation of parietal pleural invasion of lung cancers with breathhold inspiration and expiration MRI. Clin Imaging, 1999, 23(4): 227-235. doi: 10.1016/s0899-7071(99)00135-7 10631899

[24] Glazer HS, Duncan-Meyer J, Aronberg DJ, et al. Pleural and chest wall invasion in bronchogenic carcinoma: CT evaluation. Radiology, 1985, 157(1): 191-194. doi: 10.1148/radiology.157.1.4034965 4034965

[25] Imai K, Minamiya Y, Ishiyama K, et al. Use of CT to evaluate pleural invasion in non-small cell lung cancer: measurement of the ratio of the interface between tumor and neighboring structures to maximum tumor diameter. Radiology, 2013, 267(2): 619-626. doi: 10.1148/radiol.12120864 23329658

[26] Ebara K, Takashima S, Jiang B, et al. Pleural invasion by peripheral lung cancer: prediction with three-dimensional CT. Acad Radiol, 2015, 22(3): 310-319. doi: 10.1016/j.acra.2014.10.002 25542401

[27] Pennes DR, Glazer GM, Wimbish KJ, et al. Chest wall invasion by lung cancer: limitations of CT evaluation. AJR Am J Roentgenol, 1985, 144(3): 507-511. doi: 10.2214/ajr.144.3.507 3871560

[28] Uhrmeister P, Allmann KH, Wertzel H, et al. Chest wall infiltration by lung cancer: value of thin-sectional CT with different reconstruction algorithms. Eur Radiol, 1999, 9(7): 1304-1309. doi: 10.1007/s003300050837 10460363

[29] Kuriyama K, Tateishi R, Kumatani T, et al. Pleural invasion by peripheral bronchogenic carcinoma: assessment with three-dimensional helical CT. Radiology, 1994, 191(2): 365-369. doi: 10.1148/radiology.191.2.8153307 8153307

[30] Chen Y, Huang Q, Zhong H, et al. Correlations between iodine uptake, invasive CT features and pleural invasion in adenocarcinomas with pleural contact. Sci Rep, 2023, 13(1): 16191. doi: 10.1038/s41598-023-43504-0 PMC10533497 37758831

[31] Lennartz S, Le Blanc M, Zopfs D, et al. Dual-energy CT-derived iodine maps: use in assessing pleural carcinomatosis. Radiology, 2019, 290(3): 796-804. doi: 10.1148/radiol.2018181567 30644812

[32] Iizuka S, Kawase A, Oiwa H, et al. A risk scoring system for predicting visceral pleural invasion in non-small lung cancer patients. Gen Thorac Cardiovasc Surg, 2019, 67(10): 876-879. doi: 10.1007/s11748-019-01101-x 30888590

[33] Usuda K, Iwai S, Funasaki A, et al. Diffusion-weighted imaging can differentiate between malignant and benign pleural diseases. Cancers (Basel), 2019, 11(6): 811. doi: 10.3390/cancers11060811 PMC6627409 31212757

[34] Suzuki N, Saitoh T, Kitamura S. Tumor invasion of the chest wall in lung cancer: diagnosis with US. Radiology, 1993, 187(1): 39-42. doi: 10.1148/radiology.187.1.8451433 8451433

[35] Yuan M, Liu JY, Zhang T, et al. Prognostic impact of the findings on thin-section computed tomography in stage I lung adenocarcinoma with visceral pleural invasion. Sci Rep, 2018, 8(1): 4743. doi: 10.1038/s41598-018-22853-1 PMC5856785 29549366

[36] Perrella A, Bagnacci G, Di Meglio N, et al. Thoracic diseases: technique and applications of dual-energy CT. Diagnostics (Basel), 2023, 13(14): 2440. doi: 10.3390/diagnostics13142440 PMC10378112 37510184

[37] Hsu JS, Han IT, Tsai TH, et al. Pleural tags on CT scans to predict visceral pleural invasion of non-small cell lung cancer that does not abut the pleura. Radiology, 2016, 279(2): 590-596. doi: 10.1148/radiol.2015151120 26653684

[38] Qi LP, Li XT, Yang Y, et al. Multivariate analysis of pleural invasion of peripheral non-small cell lung cancer-based computed tomography features. J Comput Assist Tomogr, 2016, 40(5): 757-762. doi: 10.1097/RCT.0000000000000439 27224225

[39] Wei SH, Zhang JM, Shi B, et al. The value of CT radiomics features to predict visceral pleural invasion in ≤3 cm peripheral type early non-small cell lung cancer. J Xray Sci Technol, 2022, 30(6): 1115-1126. doi: 10.3233/XST-221220 35938237

[40] Ahn SY, Park CM, Jeon YK, et al. Predictive CT features of visceral pleural invasion by T1-sized peripheral pulmonary adenocarcinomas manifesting as subsolid nodules. AJR Am J Roentgenol, 2017, 209(3): 561-566. doi: 10.2214/AJR.16.17280 28639833

[41] Adachi H, Tsuboi M, Nishii T, et al. Influence of visceral pleural invasion on survival in completely resected non-small-cell lung cancer. Eur J Cardiothorac Surg, 2015, 48(5): 691-697; discussion 697. doi: 10.1093/ejcts/ezu515 25564209

[42] Hung JJ, Jeng WJ, Hsu WH, et al. Prognostic significance of the extent of visceral pleural invasion in completely resected node-negative non-small cell lung cancer. Chest, 2012, 142(1): 141-150. doi: 10.1378/chest.11-2552 22241763

[43] Haggar AM, Pearlberg JL, Froelich JW, et al. Chest-wall invasion by carcinoma of the lung: detection by MR imaging. AJR Am J Roentgenol, 1987, 148(6): 1075-1078. doi: 10.2214/ajr.148.6.1075 3034011

[44] Musset D, Grenier P, Carette MF, et al. Primary lung cancer staging: prospective comparative study of MR imaging with CT. Radiology, 1986, 160(3): 607-611. doi: 10.1148/radiology.160.3.2426726 2426726

[45] Akata S, Kajiwara N, Park J, et al. Evaluation of chest wall invasion by lung cancer using respiratory dynamic MRI. J Med Imaging Radiat Oncol, 2008, 52(1): 36-39. doi: 10.1111/j.1440-1673.2007.01908.x 18373824

[46] Akhurst T, Downey RJ, Ginsberg MS, et al. An initial experience with FDG-PET in the imaging of residual disease after induction therapy for lung cancer. Ann Thorac Surg, 2002, 73(1): 259-264; discussion 264-266. doi: 10.1016/s0003-4975(01)03257-x 11834020

[47] Han Y, Wang ZG, Liu SM, et al. MSCT and ^18^F-FDG PET/CT features of pleural indentation in evalution of peripheral lung cancer pleural invasion. Zhongguo Yixue Yingxiang Jishu, 2014, 30(12): 1835-1838.

